# Quantitative evaluation of diaphragmatic motion during forced breathing in chronic obstructive pulmonary disease patients using dynamic chest radiography

**DOI:** 10.3389/fnint.2022.842404

**Published:** 2022-10-05

**Authors:** Jianghong Chen, Zhaohui Zhong, Wei Wang, Ganggang Yu, Tingting Zhang, Zhenchang Wang

**Affiliations:** ^1^Department of Radiology, Beijing Friendship Hospital, Capital Medical University, Beijing, China; ^2^Department of Respiration, Beijing Friendship Hospital, Capital Medical University, Beijing, China

**Keywords:** chronic obstructive pulmonary disease (COPD), diaphragmatic motion, forced breathing, dynamic chest radiograph, prospective study

## Abstract

**Objective:**

To quantitatively evaluate the bilateral diaphragmatic motion difference during forced breathing between chronic obstructive pulmonary disease (COPD) patients and healthy individuals using dynamic chest radiography technique.

**Methods:**

This prospective study included the COPD patients (n: 96, f/m: 17/79, age: 66 ± 8 years old) and healthy individuals (n: 50, f/m: 42/8, age: 53 ± 5 years old) that underwent dynamic chest radiography with a flat panel X-ray detector system during forced breathing in a standing position. After analyzing the excursions, duration and velocity of diaphragmatic motion were automatically calculated using the postprocessing software. The parameters of diaphragmatic motion including excursion, duration, velocity, inhalation/exhalation times were assessed in all subjects for both diaphragms. The correlation between lung function parameters and diaphragmatic motion excursions were further evaluated.

**Results:**

The excursions of diaphragmatic motion in COPD patients were significantly decreased in COPD patients compared with healthy individuals during forced breathing (*P* < 0.05). The excursion in COPD patients was 35.93 ± 13.07 mm vs. 41.49 ± 12.07 mm in healthy individuals in the left diaphragm, and 32.05 ± 12.29 mm in COPD patients vs. 36.88 ± 10.96 mm in healthy individuals in the right diaphragm. The duration of diaphragmatic motion significantly decreased in COPD patients, compared with the healthy individuals (*P* < 0.05). The inhalation time in COPD patients was 2.03 ± 1.19 s vs. 2.53 ± 0.83 s in healthy individuals in the left diaphragm and 1.94 ± 1.32 s in COPD patients vs. 2.23 ± 1.21 s in healthy individuals in the right diaphragm. The exhalation time was 4.77 ± 1.32 s in COPD patients vs. 6.40 ± 2.73 s in healthy individuals in the left diaphragm and 4.94 ± 3.30 s in COPD patients vs. 6.72 ± 2.58 s in healthy individuals in the right diaphragm. The peak velocity of diaphragmatic motion showed no significant difference between COPD and healthy groups. The excursions of bilateral diaphragmatic motion showed moderate correlation with FEV1/FVC (*r* = 0.44, *P* < 0.001). Multi-linear regression analysis showed that the excursions of bilateral diaphragm are significantly associated with COPD occurrence (*P* < 0.05).

**Conclusion:**

The excursions and duration of diaphragmatic motion during forced breathing are significantly decreased in COPD patients, compared with healthy individuals. Our study showed that precise bilateral diaphragmatic motion activity can be evaluated by dynamic chest radiography.

## Introduction

Chronic obstructive pulmonary disease (COPD) is a chronic, preventable and treatable, respiratory condition, characterized by shortness of breath, cough and recurrent exacerbations (Lozano et al., 2012). In this disorder, anomalies in the small airways of the lungs limit the airflow in and out of the lungs. Different risk factors and processes have been reported as main causes of narrowing airways including destruction of parts of the lung, mucus blocking the airways, and inflammation and swelling of the airway lining (Morgan and Summer, 2008). COPD is the third leading cause of mortalities worldwide, causing 3.23 million deaths in 2019 (Stanaway et al., 2018). In China, COPD is among the four most common chronic diseases. With a prevalence of 13.7% in Chinese populations over 40 years of age and affecting approximately 100 million people, COPD is currently a major public health issue that imposes significant economic burden in China (Wang et al., 2018). This disorder causes persistent and progressive respiratory symptoms, including difficulty in breathing, cough and phlegm production. COPD is a systematic chronic disease that affects both men and women and the whole body is involved in the disorder. COPD-associated symptoms such as inflammatory cytokines, insufficient oxygen supply of tissue, electrolyte disturbance and long-term use of steroid hormone will severely affect the skeleton muscles throughout the body (Barreiro and Gea, 2015; Jaitovich and Barreiro, 2018) including diaphragm, which will further cause diaphragm fatigue and contraction weakness (Charususin et al., 2018). Pulmonary ventilation is highly dependent on diaphragm mobilization, which attributes for nearly 80% of the pulmonary ventilation. Therefore, accurate assessment of diaphragm function helps to evaluate the progression of COPD and can provide guidance for the clinical treatment of COPD.

Precision medicine for therapy and diagnosis of disorders has dramatically progressed during the recent years (Agustí et al., 2017; Sidhaye et al., 2018). Precision medicine for COPD has also witnessed significant development recently. Recent findings from modeling, imaging and molecular studies on the heterogeneous and complicated nature of COPD have identified different clinical and inflammatory phenotypes that highlight the need for shifting the focus of health management strategy from prototypic disease labels toward targeted therapies (Agustí et al., 2017; Wouters et al., 2017; Sidhaye et al., 2018; Franssen et al., 2019; Ulrik et al., 2020).

In brief, precision medicine approach aims to identify and connect the right treatment to the right patient, meanwhile minimizing the risk of adverse effects. Recent studies have identified several treatable features for different types of COPD including pulmonary, extra-pulmonary, psychological, and environmental traits (Hersh, 2019; Dennett et al., 2021). The next step is identifying and quantifying physiological features, molecular pathways and underlying mechanisms of these treatable traits through innovative modeling or detection techniques and then developing noble reliable point-of-care biomarkers to predict and/or differentiate responders from non-responders to targeted therapies or precision medicine (Wouters et al., 2017; Cazzola et al., 2018; Sidhaye et al., 2018).

In this study, we used a flat panel X-ray detector, which can obtain dynamic chest radiography of breathing motion in patient in the standing position. Dynamic radiographs are more conducive to intuitively diaphragmatic motion, compared with the static images of conventional chest radiographs. More importantly, post-processing of the image obtained from this type of detector, further provides quantitative parameters such as the excursions, durations and peak velocity of diaphragm longitudinal motion, which is conducive to precise evaluation of the diaphragmatic motion in patient. Recently, several relevant studies reported the application of this device to evaluate the diaphragmatic motion, which reported significant outcomes, but these studies are limited to single-center and small-sample of COPD patients. To the best knowledge of the authors, this study is the first application of the dynamic chest radiography to quantitative assessment of diaphragmatic motion in COPD patients in China and will further confirmed its diagnostic value of COPD.

## Materials and methods

### Clinical materials

All experimental procedures of this study were approved by the institutional review board and Ethics Committee of Beijing Friendship Hospital affiliated to Capital Medical University, Beijing, China (Ethics code: 2018-P2-086-01) that were in accordance with the regulations and guidelines of the studies on human subjects set by the Declaration of Helsinki (General Assembly of the World Medical Association, 2014).

Written informed consent forms were obtained from all patients in the study. The subjects were continuously enrolled from June 2018 to June 2019. The inclusion criteria for COPD patients included: 1. Age range of 40–75 years old, 2. Diagnosis of stage I-IV COPD according to the 2017 Global Initiative for Chronic Obstructive Lung Disease (GOLD) guideline, 3. Current smoking or smoking history for the last 20 years, 4. Written consent for participating in the study. Exclusion criteria included: 1. Patients with bronchial asthma, 2. Patients with other diffuse pulmonary disease, 3. Patients with acute pulmonary infection or pulmonary space-occupying lesions, 4. Patients with obvious pleural or chest wall lesion, 5. Patients with lung surgery history. A total of 96 COPD patients were enrolled in the study, including 79 male (82.29%) and 17 female (17.71%), average age 67 ± 7 years old. The inclusion criteria of healthy individuals included 1. Age range 40–75 years of age, 2. Healthy condition before the study and lack of any history of heart and lung disease, 3. Normal lung function (FEV1/FVC > 70%), 4. No smoking history, 5. Written consent form on participating in the study. The individuals including 8 males (16.00%) and 42 females (84.00%), average age of 53 ± 5 years old were enrolled in the study. The height and body weight of all participate were recorded and body mass index BMI (weight/height ^2^) were calculated.

### Device and data analysis

A flat panel dynamic X-ray digital radiography apparatus (Konica Minolta Inc., Tokyo, Japan) was used in this study. Before radiography, the participants were taught to learn forced inhalation to reach maximum inspiratory capacity (IC) and followed by forced exhalation to reach maximum expiratory capacity. Subjects were in a post-anterior standing position, with scanning parameters of 100 kV tube voltage, 50 mA tube current, 388 × 388 μm, 1,024 × 768 matrix and with an emitting pulse at 15 frames/second. The X-ray were set to pulse emission to reduce the radiation dose, exposure time were set to 15 s with a radiation dose of approximately 0.34 mSv for each participant.

**FIGURE 1:**
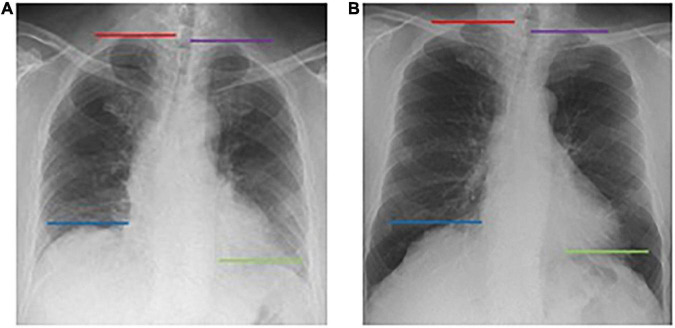
The Kinetic Analysis Tool™ automatically recognizes the bilateral pulmonary apexes and diaphragm dome. Dynamic X-ray working station automatically marked the bilateral diaphragm dome (right diaphragm: blue line; left diaphragm: green line) and lung apex (right lung: red line; left lung: purple line) in both end exhalation **(A)** and end inhalation **(B)**, and tracing the position change with breathing.

**FIGURE 2:**
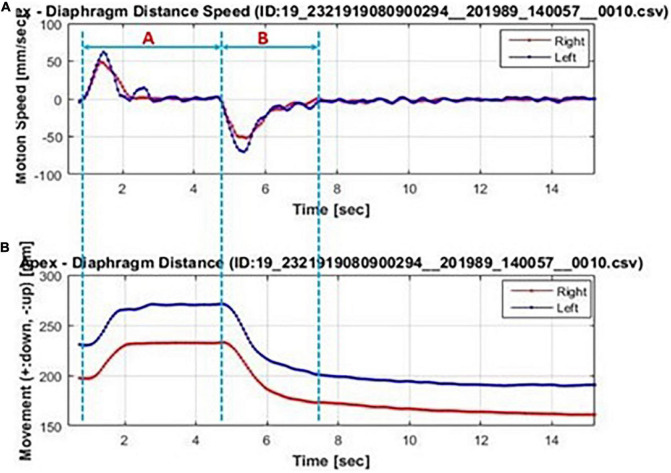
Bilateral diaphragmatic motion excursions and velocity vs. time curve automatically generated by the Kinetic Analysis Tool™. Bilateral diaphragm (left diaphragm: blue line; right diaphragm: red line) motion velocity-time curve **(A)** and motion excursions-time curve (B). The forced inhalation stage was marked with blue dotted line A and the forced exhalation stage are marked with blue dotted line B. In **(A)**, the corresponding diaphragm velocity were of positive value in stage A and of negative value in stage B. The wave crest and trough representant the diaphragm maximum motion velocity in inhalation and exhalation, respectively. In **(B)**, the maximum diaphragmatic motion excursions were representant as the difference value between maximum value in stage A and the minimum value in stage B.

The obtained dynamic chest radiographs were transmitted to the assorted workstation and then went under analysis using the Kinetic Analysis Tool assorted to the workstation. This software can automatically recognize and mark the bilateral lung apex and the edges of diaphragm (Figure 1). A radiologist with over 15 years of diagnosis chest radiography experience will select the end-expiratory images and correct the highest point of bilateral diaphragm and positions of bilateral lung apexes which will be marked automatically by the software. Then, the motion trail was automatically traced by the software and the time curve of vertical dimension from diaphragm to lung apexes were painted and corresponding diaphragmatic motion velocity vs. time curve (Figure 2). The value of any point on the curves can be displayed, the excursions, durations and peak velocities of bilateral diaphragmatic motion were obtained based on the values were shown on the curves.

### Statistical analyses

Statistical analyses were performed using Statistical Package for Social Sciences (SPSS) (IBM SPSS Inc., Chicago IL, Windows version 20). Continuous data were employed with normal distribution test. The variables with normal distribution were presented as means ± standard deviation (s) and variables with skewed distribution were presented in median (Inter Quartile Range, IQR). For comparative analysis of the COPD patients and healthy individuals, *t*-test was used for normal distribution data and non-parametric test (*Mann-Whitney U*) was used for skewed distribution data, and *Pearson* Chi-square test was used for enumeration data. The correlations between pulmonary function parameters and diaphragmatic motion excursions were analyzed using *Pearson* correlation analysis for normal distribution data and *Spearman* correlation analysis for skewed distribution data. For all correlation tests, *r* = 0.10∼0.39 was considered a weak correlation; *r* = 0.40∼0.69 a medium correlation and *r* = 0.70∼1.00 was considered a strong correlation. Multiple linear regression was further used to analyze the correlation between bilateral diaphragmatic motion excursions and the COPD occurrence after correction for the gender, age and BMI. In all statistical analyses the *P* < 0.05 was considered as significant difference.

## Results

Basic clinical characteristics of the 96 COPD patients and 50 healthy individuals are presented in Table 1. There were significant differences in age, gender and height but no significant difference in BMI between the COPD and the healthy groups. Significant difference in IC, which reflects pulmonary capacity, was found between COPD patients and healthy individuals. Pulmonary ventilation parameters such as forced vital capacity (FVC), forced expiratory volume in on second (FEV1), and FEV1/FVC were significantly different between COPD patients and healthy individuals.

**TABLE 1:** Basic clinical characteristics and pulmonary function assessment of COPD patients and healthy individuals.

	COPD patients (*n* = 96)	Healthy individuals (*n* = 50)	Statistics *t* or χ^2^-value	*P*-value
Age (year)	66.21 ± 8.31	53.44 ± 5.38	9.84	<0.001
Gender (male/female)	79/17	8/42	60.00	<0.001
Height (cm)	167.50(6.75)	160.00(7.25)	–4.81	<0.001
Weight (kg)	68.94 ± 12.27	66.98 ± 9.24	0.99	0.322
BMI (Kg/m^2^)	24.69 ± 3.94	25.65 ± 2.94	–1.52	0.131
**Lung function**				
FEV1% predicted value (%)	58.18 ± 21.49	110.60 ± 21.44	25.37	<0.001
FEV1/FVC% predicted value (%)	53.56 ± 11.11	79.87 ± 5.32	–19.34	<0.001
FVC% predicted value (%)	83.76 ± 20.00	102.75 ± 14.08	–6.66	<0.001
IC% predicted value (%)	79.92 ± 25.07	105.10 ± 15.18	–7.47	<0.001

The bilateral diaphragmatic motion activity during forced breathing in both groups were recorded and sorted in Table 2. The diaphragmatic motion excursions and durations of inhalation and exhalation during forced breathing are significantly decreased in COPD patients, compared with healthy individuals (*P* < 0.05), but no significant difference of diaphragmatic motion velocity is observed between the two groups. The motion excursions of left diaphragm are significantly higher than the right side in both COPD and healthy groups (*P* = 0.025 in COPD patients and *P* = 0.048 in healthy individuals). In COPD patients, the motion velocity of left diaphragm is significantly higher than the right side in both inhalation stage (*P* = 0.005) and exhalation stage (*P* = 0.006), but there is no significant difference between left and right sides in healthy individuals (*P* = 0.083 in inhalation stage and *P* = 0.109 in exhalation stage).

**TABLE 2:** Comparative analysis of bilateral diaphragmatic motion activity during forced breathing in COPD patients and healthy individuals.

		COPD patients (*n* = 96)	Healthy individuals (*n* = 50)	Statistics *t*- or *Z*-value	*P*-value
Diaphragm longitudinal motion excursions (mm)	Left	35.93 ± 13.07	41.49 ± 12.07	–2.501	0.014
	Right	32.05 ± 12.29	36.88 ± 10.96	–2.334	0.021
Diaphragm descending time during inhalation (s)	Left	2.03(1.19)	2.53 ± 0.83	–2.45	0.014
	Right	1.94(1.32)	2.23(1.21)	–2.62	0.009
Diaphragm ascending time during exhalation (s)	Left	4.77(3.25)	6.40 ± 2.73	–2.42	0.016
	Right	4.94(3.30)	6.72 ± 2.58	–2.93	0.003
Peak diaphragmatic motion velocity in inhalation (mm/s)	Left	38.50 ± 14.42	37.94 ± 12.51	0.232	0.817
	Right	32.98 ± 12.70	33.64 ± 12.05	–0.301	0.764
Peak diaphragmatic motion velocity in exhalation (mm/s)	Left	34.46 ± 14.68	37.00(23.88)	–1.38	0.168
	Right	25.50(19.38)	29.25(18.63)	–1.86	0.063

**FIGURE 3:**
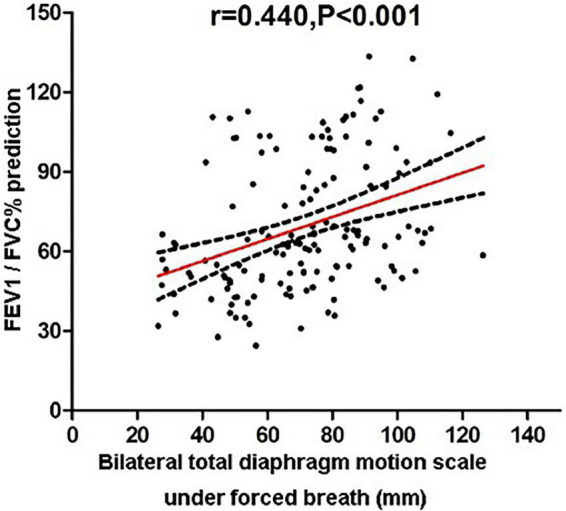
Correlation analysis of FEV1/FVC% predicted values and bilateral total diaphragmatic motion excursions during forced breathing.

Then, the correlations between pulmonary function parameters and bilateral diaphragm longitudinal motion excursions were analyzed (Table 3). The FEV1/FVC% predicted values showed the highest correlation with the diaphragmatic motion excursions among all the lung function parameters, which was of moderate correlation (Figure 3).

**TABLE 3:** Correlation analysis of pulmonary function parameters and bilateral total diaphragmatic motion excursions during forced breathing.

Pulmonary function parameter	Diaphragmatic motion excursions	
	
	*r*	*P*
FEV1% predicted values	0.364	<0.001**
FEV1/FVC% predicted values	0.440	<0.001**
FVC% predicted values	0.245	0.016*
IC% predicted values	0.325	0.002**

*P < 0.05, **P < 0.01.

Finally, multiple linear regression analysis was conducted to analyze the correlations between bilateral diaphragmatic motion excursions and the COPD occurrence after corrected for the gender, age and BMI. The results showed that COPD occurrence (*P* = 0.003) and FEV1% predicted values (*P* = 0.001) are independent influencing factors of left diaphragmatic motion excursions. Moreover, COPD occurrence (*P* = 0.010) and FEV1% predicted values (*P* = 0.002) are independent influencing factor of right diaphragmatic motion excursions (Table 4).

**TABLE 4:** Multiple linear regression correction analysis of bilateral diaphragmatic motion excursions and the COPD progress after base line correction.

Variables	Left diaphragm longitudinal motion excursions (mm)			Right diaphragm longitudinal motion excursions (mm)		
		
	Regression coefficient	*t*	*P*	Regression coefficient	*t*	*P*
COPD occurrence (patient vs. control)	–15.508	–3.073	0.003**	–12.343	–2.615	0.010*
Gender	1.453	0.534	0.594	1.035	0.407	0.685
Age	0.056	0.358	0.721	–0.062	–0.423	0.673
BMI	0.550	1.954	0.053	0.498	1.893	0.060
FEV1% prediction (%)	0.192	3.276	0.001**	0.171	3.105	0.002**

*P < 0.05, **P < 0.01.

## Discussion

The contraction and dilation of diaphragm are the main driving force of pulmonary ventilation. In healthy people, the bilateral diaphragm is smoothy and dome-shaped with excursions of approximately 2∼3 cm during tidal breathing and 3∼6 cm during deep breathing (Yamada et al., 2017a). In this study, all subjects were in standing position during dynamic chest radiography with a flat panel X-ray detector, which maintains the normal daily position of patients. Therefore, the data acquired by dynamic chest radiography is more accurate than other imaging methods such as computed tomography (CT) and magnetic resonance imaging (MRI), in which subjects are in a supine position. The enroll criteria for the COPD patients followed the 2017 GOLD, that is, the patient has persistent respiratory symptoms, airflow limitation, and FEV1/FVC < 0.7 on pulmonary function tests after giving bronchodilators, which indicates the patient has persistent airflow limitation. The latest 2020 GOLD Guideline still maintains the diagnostic criteria of 2017 GOLD Guideline (Global Initiative for Chronic Obstructive Lung Disease, 2020).

As the airflow limitation and air trapping in COPD patients causes significantly increased respiratory load. It is reported that the respiratory load increased 5∼10 times in COPD patients compared with healthy individuals (MacIntyre and Leatherman, 1989). What’s more, excessive expansion of lung tissue causes diaphragmatic depression, which leads to shortened initial contraction length of diaphragm, reduces diaphragm tension and affecting the respiratory movements. The inflammatory cytokines produced by COPD severely affect skeletal muscles throughout the whole body, including the diaphragm. In addition, tissue insufficient oxygen supply, electrolytic disorder further damages the diaphragm (Barreiro and Gea, 2015; Yamada et al., 2017b; Charususin et al., 2018; Jaitovich and Barreiro, 2018; Lee and Kim, 2019) and will finally cause diaphragm fatigue or even respiratory failure, which will severely reduce patient’s life quality. Previous studies mainly focused on evaluation of diaphragm morphology or functional imaging (Kharma, 2013; Koo et al., 2018; Do Nascimento and Fleig, 2020). Traditional X-ray imaging is a preliminary test for evaluating diaphragm morphology alteration (Chetta et al., 2005). Further CT test will identify a variety of causes of abnormal morphology and position of diaphragm (Nason et al., 2012), for example, diaphragmatic elevation caused by subphrenic lesions or unilateral diaphragmatic paralysis caused by malignant lesions invading phrenic nerve. Dynamic MRI test can further evaluate diaphragm function, but the MRI is technically complicated and require high operation skills (Kiryu et al., 2006). Ultrasonography is the most common imaging modality to evaluate the morphology and function of diaphragm in clinic. It is simple and free of ionizing radiation and it can be used to measure both the direction and excursions of diaphragmatic motion (Connolly and Mittendorfer, 2016; Corbellini et al., 2018). The dynamic X-ray radiography used in this study has its own unique advantages compared with the abovementioned imaging methods. First, the equipment requires low patient compliance, especially for patients who cannot hold their breath long enough to complete a CT scan or for patients who cannot tolerate long scanning time of MRI, this equipment can also be used to evaluate the diaphragmatic motion in COPD patients. Then, this equipment can be used to monitor the morphological changes, motion excursions and motion consistency of bilateral diaphragms thoroughly. More importantly, it can provide quantitative data such as diaphragmatic motion excursions and velocity, which are conducive to a precise diagnosis.

In our preliminary study, we analyzed diaphragmatic motion status of COPD patients during tidal breathing. The results showed that the diaphragmatic longitudinal motion excursions in COPD patients are significantly longer than healthy people, and this finding is consistent with the previous report by Yamada et al. (2017b). On this basis, the present study further analyzed the diaphragmatic motion status of COPD patients during forced breathing. The results showed that the diaphragmatic motion excursions in COPD patients are significantly shorter than healthy individuals, and this finding is consistent with the previous report by Hida et al. (2019). The different results of diaphragmatic motion during different breathing condition reflect that COPD pathological changes affect the diaphragmatic motion activity, which is mentioned above. Forced breathing can better reflects the motion state of diaphragm and pulmonary function, compared with tidal breathing. During forced breathing the diaphragm can no longer maintain its compensatory ability of diaphragm as in tidal breathing. In our study, the bilateral diaphragmatic motion velocity shows no significant difference between COPD patients and healthy individuals in both inhalation and exhalation phase. However, Hida et al., 2019 reported that the left diaphragmatic motion velocity is significantly lower than healthy individuals in inhalation phase, that might be because of the difference of the sample size of the study subjects and patient’s overall lung function. Hida et al. (2019) enrolled only 31 COPD patients, so their results need further validation from multi-central studies with a larger sample size. We further compared the difference in bilateral diaphragmatic motion during forced breathing between COPD patients and healthy individuals. Our results showed that the left diaphragmatic motion excursions are significantly bigger than the right side in both groups and the maximum motion velocity of left diaphragm is significantly higher than the right side. This difference could be attributed to the restriction caused by liver over the active contraction and descending of right diaphragm.

Univariate analysis showed that the diaphragmatic motion excursion during forced breathing was of moderate correlation with FEV1/FVC (*r* = 0.440). Because the enrolled COPD patients were of elderly age group and predominantly male, and we failed to enroll enough age- and gender-matched healthy individuals with matched age and gender. After correction for the gender, age and BMI, multiple linear regression analysis showed that COPD occurrence and FEV1% predicted values is an independent influence factor of bilateral diaphragmatic motion excursions. Hida et al. (2019), using the same dynamic X-ray device as we do reported that in COPD patients the diaphragmatic motion excursions are correlated to VC and FEV1 during forced breathing using the same. Yamada et al. (2017b) reported that COPD occurrence and BMI are independent influence factor of diaphragmatic motion excursions. Therefore, dynamic X-ray device can be used as an auxiliary diagnosis to evaluate the lung function and degree of diaphragm lesions in COPD patients, especially for those with poor pulmonary function or unable to complete the pulmonary function test due to failure to comprehend and cooperate with the instructions. What’s more, using the post-processing function of the device we can also obtain the functional images of pulmonary ventilation and pulmonary circulation from the images of dynamic chest films. The clinical applications of this device needed further development and utilization.

This study has some limitations. First, it was a single-center trial. Therefore, studies with larger sample size are needed to validate the results. Second, the dynamic digital radiography system is a newly developed device. The obtained radiographs only contained anteroposterior position information but lacked lateral position information, so further improvement is needed to provide more objective data for clinical study. Third, there were relatively few GOLD grades III or IV patients enrolled in this study, no further grading study has been carried out, so we still need to increase the sample size to further improve the assessment.

## Conclusion

In conclusion, the objective and accurate quantitative data of diaphragmatic motion can be obtained using dynamic chest radiography, which is a valuable examination method to evaluate the condition of COPD patients.

## Data availability statement

The original contributions presented in this study are included in the article/supplementary material, further inquiries can be directed to the corresponding author.

## Ethics statement

All experimental procedures of this study were approved by the Institutional Review Board and Ethics Committee of Beijing Friendship Hospital affiliated to Capital Medical University, Beijing, China (Ethics code: 2018-P2-086-01) that were in accordance with the regulations and guidelines of the studies on human subjects set by the Declaration of Helsinki. The patients/participants provided their written informed consent to participate in this study.

## Author contributions

JC and ZZ conducted the analytical part, wrote the first version of the manuscript, and finalized the manuscript. WW, GY, and TZ collected the data. ZW supervised the study. ZZ conceived and coordinated the study, and critically evaluated the data. All authors read and approved the final manuscript.
